# Caffeine differentially alters cortical hemodynamic activity during working memory: a near infrared spectroscopy study

**DOI:** 10.1186/s13104-015-1491-3

**Published:** 2015-10-01

**Authors:** Urs Heilbronner, Hermann Hinrichs, Hans-Jochen Heinze, Tino Zaehle

**Affiliations:** DZNE, German Center for Neurodegenerative Diseases, Otto-von-Guericke University, Magdeburg, Germany; Institute of Psychiatric Phenomics and Genomics, Ludwig-Maximilians-University, Munich, Germany; LIN, Leibniz Institute for Neurobiology, Magdeburg, Germany; Department of Neurology, Otto-von-Guericke University, Magdeburg, Germany

**Keywords:** NIRS, Caffeine, n-back, 2-back, Working memory, Adenosine, BOLD, WM

## Abstract

**Background:**

Caffeine is a widely used stimulant with potentially beneficial effects on cognition as well as vasoconstrictive properties. In functional magnetic imaging research, caffeine has gained attention as a potential enhancer of the blood oxygenation level-dependent (BOLD) response. In order to clarify changes of oxy- and deoxyhemoglobin (HbO and HbR) induced by caffeine during a cognitive task, we investigated a working memory (WM) paradigm (visual 2-back) using near-infrared spectroscopy (NIRS).

**Results:**

Behaviorally, caffeine had no effect on the WM performance but influenced reaction times in the 0-back condition. NIRS data demonstrate caffeine-dependent alterations of the course of the hemodynamic response. The intake of 200 mg caffeine caused a significant decrease of the HbO response between 20 and 40 s after the onset of a 2-back task in the bilateral inferior frontal cortex (IFC). In parallel, the HbR response of the left IFC was significantly increased due to caffeine intake.

**Conclusions:**

In line with previous results, we did not detect an effect of caffeine on most aspects of behavior. Effects of caffeine on brain vasculature were detected as general reduction of HbO. Neuronal effects of caffeine are reflected in an increased concentration of HbR in the left hemisphere when performing a verbal memory task and suggest influences on metabolism.

## Background

Caffeine is one of the most widely consumed stimulants worldwide. Ingredient of a variety of foods and beverages, low doses of caffeine generally produce feelings of well-being and increased alertness in humans. Although there are reports about caffeine facilitating cognitive functions (e.g. [1–3], these psychological effects have been attributed to increased arousal and overcoming fatigue rather than to a direct enhancement of specific cognitive functions (reviewed by [4]). The major pharmacological action of the stimulant is that of a nonspecific adenosine receptor antagonist (for review see [5]). Since adenosine receptors display a general inhibitory effect on neural activity, the receptor antagonism of caffeine has stimulant properties through disinhibitory mechanisms. However, caffeine further acts as a vasoconstrictor that causes reductions in cerebral blood flow (CBF) [5–7]. In fMRI research, the latter effect has been discussed as a method to boost the blood oxygenation level-dependent (BOLD) contrast [8]. In humans, a dose of 200 mg caffeine can decrease the resting-state CBF to approximately 30 % [9, 10]. As the magnitudes of vascular responses to stimuli remain identical [11], this results in a maximized BOLD contrast. However, results on the BOLD-boosting capability of caffeine are far from being consistent and are discussed controversially in the literature [12, 13].

The BOLD signal is a complex function of changes in neural activity, oxygen metabolism, cerebral blood volume, CBF and further physiological parameters [14]. Among other factors, the relation of oxy- and deoxyhemoglobin (HbO and HbR) are critical determinants of the BOLD response. As concentrations HbO and HbR can be measured by near-infrared spectroscopy (NIRS), we used this technique to investigate components underlying the BOLD response. NIRS is an optical technique that is based on absorption of light in the near-infrared spectrum [15–17] and can be used for functional brain mapping (e.g. [18–23]. NIRS is able to measure relative cortical concentrations of both HbO and HbR independently (for review see [24]).

To investigate the above mentioned issue in a standard cognitive paradigm, we used the 2-back task to investigate effects of caffeine. This task assesses working memory (WM; [25]), which is the ability to hold and manipulate information for a short amount of time. NIRS has been successfully used to measure brain activity associated with WM [26], demonstrating a linear increase of hemodynamic responses with WM load [27].

## Methods

### Participants

Eleven subjects participated in the experiment. Data from one subject were excluded due to excessive NIRS signal drifts and two malfunctioning NIRS channels. The remaining 10 participants (7 female) were 29.1 ± 1.6 (mean ± SEM; range 19–34) years old. All participants were classified as moderate caffeine consumers [28], with a averaged consumption of 2-3 cup of coffee per day. All experiments were carried out in the morning and participants were instructed to abstain from drinking coffee the morning before the experiment. All subjects reported to have complied with this instruction. Ethical approval was obtained prior to the study from the ethics committee of the University Magdeburg and all participants gave written informed consent before participation.

### Experimental procedure

At the start of each experimental session, the NIRS optode grid (see below) was positioned on the subject’s forehead. During the experiment proper, participants sat in a comfortable chair in front of a computer monitor and performed the task in a dimly lit room. They were instructed in written form. Subjects saw a stream of white single letters (A, B, C, D or E) presented for 0.5 s each on a gray background with a stimulus onset asynchrony of 2 s. Stimulus presentation was controlled by the Presentation software (Neurobehavioral Systems, USA). Stimuli were presented blockwise with a total of 15 stimuli per block. The inter-block interval was varied randomly between 30 and 45 s. In a 2-back condition, subjects were asked to press a button with their index finger if the penultimate stimulus had been identical with the present stimulus or otherwise to press the button with the middle finger of their dominant hand. In a control 0-back condition, participants were instructed to respond with their index finger to all letter stimuli. The order of 0-back and 2-back blocks was pseudorandomized so that not more than 2 identical conditions followed each other. A brief practice sequence was given before the actual test.

Each participant underwent two consecutive NIRS scanning sessions, one control and one experimental session. The control session always preceded the experimental session to avoid carry-over effects (sequential paradigm). After completing eight 0-back blocks and eight 2-back blocks, the NIRS recording was paused and participants ingested a tablet containing 200 mg of caffeine. After a 30 min delay, during which the optode grid remained in place, they performed the same experimental task again. During one complete session, a total of 54 targets were presented along with 186 lures in the 2-back condition, each block containing 3 or 4 targets.

### Near-infrared spectroscopy

**Fig. 1:**
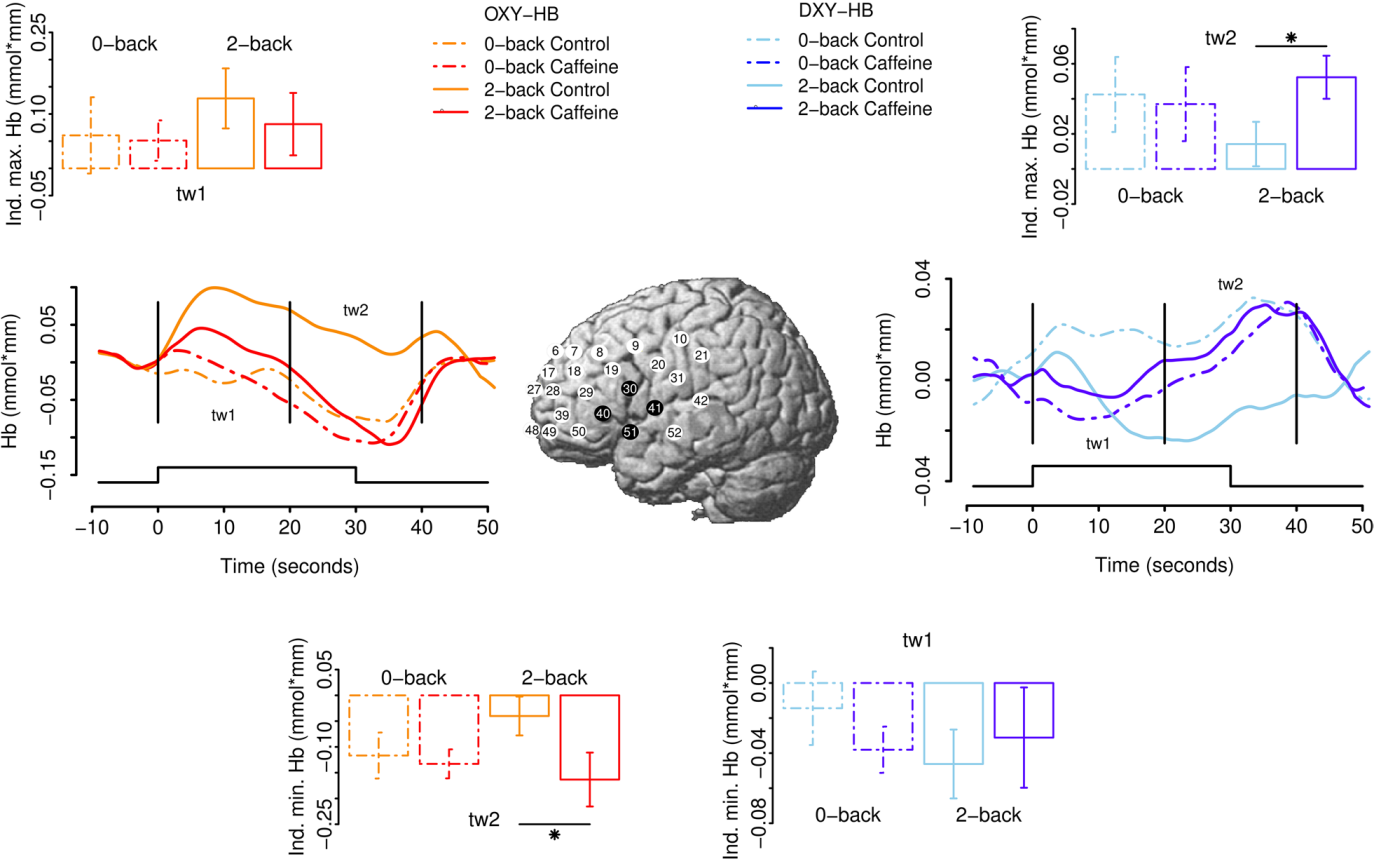
Effects of caffeine on* left* hemisphere cortical hemodynamics. The *inset* shows mean channel positions (averaged over subjects), visualized on the Montreal Neurological Institute (MNI) template brain. *Black colored positions* indicate the channels that were averaged to form the ROI. The *left*/*right line graphs* show time courses of relative concentrations of HbO/HbR in different experimental conditions. *Vertical bars* indicate the boundaries of time windows 1 and 2 (tw1 and tw2, respectively). The stimulation duration is indicated in the *lower part* of the* graphs*. The *bar graphs* above and below the *line graphs* depict mean individual maximum/minimum responses during the different time windows (see labels). The *bar graphs* depict mean HbO of the individual maximum response in tw1 (*upper left graph*), mean HbO of the individual minimum response in tw2 (*lower left graph*), mean HbR of the individual minimum response in tw1 (*lower right graph*), and mean HbR of the individual maximum response in tw2 (*upper right graph*)

**Fig. 2:**
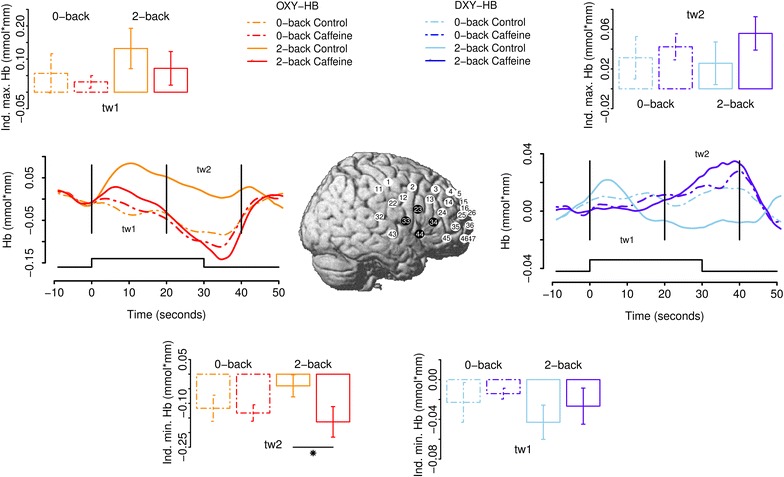
Effect of caffeine on* right *hemisphere cortical hemodynamics. The *inset* shows mean channel positions (averaged over subjects) visualized on the Montreal Neurological Institute (MNI) template brain. *Black colored positions* indicate the channels that were averaged to form the ROI. The *left*/*right line graphs* show time courses of relative concentrations of HbO/HbR hemoglobin in different experimental conditions. *Vertical bars* indicate the boundaries of time windows 1 and 2 (tw1 and tw2, respectively). The stimulation duration is indicated in the *lower part* of the* graphs*. The *bar graphs* above and below the *line graphs* depict mean individual peak responses during the different time windows (see labels). The *bar graphs* depict mean HbO of the individual maximum response in tw1 (*upper left graph*), mean HbR of the individual minimum response in tw2 (*lower left graph*), mean HbR of the individual minimum response in tw1 (*lower right graph*), and mean HbR of the individual maximum response in tw2 (*upper right graph*)

We recorded the concentration of HbO and HbR at a sampling rate of 10 Hz using Hitachi’s ETG-4000 Optical Topography System (Hitachi Medical Systems, Germany) which uses a modified Beer-Lambert law to calculate hemoglobin concentrations. Thirty-three optodes were placed on the subject’s forehead from which 52 channels were recorded. Positioning of the optode grid was performed such that the middle optode of the most inferior row on the 3 × 11 optode grid was located on the point Fpz of the international 10/20 EEG system. The distances between the optode grid and both preauricular points were kept equivalent. One drawback with this approach is, as distances between individual optodes remain constant, the scalp area under which the content of HbO and HbR are assessed can be quite variable and thus complicate interpretation of results. To ameliorate this problem, we used the ETG-4000’s built-in 3D digitizer and obtained real-world coordinates of each optode position for each individual subject [29]. Subsequently, we transformed these coordinates to the Montral Neurological Institute (MNI) framework using the toolbox of NFRI functions [30] included in the software suite NIRS-SPM [31] to be able to provide the mean channel position on the MNI brain (see insets of Figs. 1, 2). The ETG-4000 software was used for averaging hemoglobin responses to single task blocks for each subject. Data were subsequently analyzed by the R software (see below).

### Data analysis

We used the software package R (version 2.13.1, R Development Core Team, 2009) for all statistical comparisons and graphs. Signal detection theory (SDT; [32]) statistics (d′ and c) were computed for performance data in the 2-back condition. The function of Pallier [33] for R was used to compute d’. In cases in which the hit rate was equal to 1.0 or the false alarm rate was 0, we standard corrected these statistics using the formulas 1 − 1/(2 × [total number of targets]) or 1 − 1/(2 × [total number of lures]), respectively. Raw NIRS data were converted to hemoglobin concentrations, baseline corrected using the 9 s prior to the task, smoothed with a 5 s moving average, low/high-pass filtered with cutoff frequencies of 0.5/0.001 Hz, respectively, and averaged for each condition and individual subject using the ETG-4000 software package. For each individual subject, NIRS signals were averaged over all task blocks separately for each experimental condition. To detect influences of caffeine, we combined four channels on each hemisphere over the left and right inferior prefrontal cortices (PFCs) that showed clear task effects (2-back > 0-back) and averaged these to form two regions-of-interest (ROIs; see insets of Figs. 1, 2). To account for individual differences in peak vascular responses, after visual inspection of the course of the hemodynamic response, we analyzed the maximum or minimum hemodynamic response (HbO/HbR) in each of two time windows: during the first 20 s of the trial period (time window 1; tw1) and in the following 20 s (time window 2; tw2). Time window 2 encompassed 10 s post-task to capture hemodynamic changes induced late in the task period. For all statistical comparisons, we used non-parametric paired Wilcoxon signed rank tests to evade problems concerning normal distribution of data. Briefly, this procedure determines the ranks of the absolute difference between two conditions and, subsequently, restores the signs of these differences. It is then determined whether the ranks are distributed equally on either side of 0. The reported test statistic V is the sum of ranks given to the differences with positive sign, which also determines the sum of ranks assigned to the differences with negative sign.

## Results

### WM performance

Behaviorally, caffeine did not alter WM processing. In the 2-back conditions, statistics revealed no effects of the stimulant on SDT parameters (d′: no caffeine: 2.64 ± 0.25; caffeine: 2.91 ± 0.26, V = 13, p = 0.160; c: no caffeine: 0.14 ± 0.08; caffeine: 0.23 ± 0.07, V = 14, p = 0.193). Neither did caffeine influence RTs in the 2-back conditions (no caffeine: 671 ± 66 ms; caffeine: 631 ± 69, V = 37, p = 0.098). However, caffeine decreased RTs in the 0-back conditions (no caffeine: 325 ± 22 ms; caffeine: 293 ± 13 ms, V = 43, p = 0.012).

### Hemodynamics

Results are presented in Fig. 1 for the left and Fig. 2 for the right hemisphere. The effects of caffeine were analyzed separately for tw1 and tw2, the WM-load conditions (0-back, 2-back) and HbO and HbR. In the 2-back condition in tw2 (20–40 s after stimulus onset), caffeine (compared to the 2-back “no caffeine” condition) resulted in a significant decrease in HbO level (V = 50, p = 0.020) and a corresponding increase in HbR level (V = 5, p = 0.020) in the left hemisphere. In the right hemisphere, caffeine (2-back condition; tw2; compared to the 2-back “no caffeine” condition) caused a significant decrease in the HbO level (V = 50, p = 0.020), without HbR effect (V = 17, p = 0.322). All other comparisons did not reach statistical significance.

## Discussion

To the best of our knowledge, this is the first NIRS study investigating the effects of caffeine on the time course of hemoglobin concentrations during a verbal WM-task. Our data show that behaviorally, caffeine did not affect performance in the 2-back WM task, while NIRS data demonstrate caffeine dependent alterations of the course of the hemodynamic response.

### Performance data

The beneficial effects of caffeine on cognitive performance are still controversial. In accordance with a former study [34], we found no significant effect of caffeine in the 2-back task performance. There are, however, other studies reporting caffeine-related modulation of cognition, mood, and vigilance (e.g. [35, 36]). These discrepancies might be attributed to different personality traits of subjects [37], differences in the specific cognitive task or the dependent variable investigated. Also, the lack of effects on SDT statistics may stem from low statistical power due to the small number of subjects investigated. Significant facilitating effects of caffeine were observed on RTs in the 0-back condition and might be interpreted as caffeine’s effects on “a more direct and specific ‘perceptual-motor’ speed or efficiency factor” [4]. Due to the application of a sequential paradigm in the present study, training related RT improvements in the cognitive low load condition or effects of motivation cannot be excluded. In summary, the present study does not find evidence for an effect of caffeine on working memory.

### NIRS data

The present data clearly demonstrate caffeine-related alterations of the underlying hemodynamic activity. The localizations of hemoglobin concentration changes in the identified brain areas, collapsed in our ROIs, are in good agreement with other NIRS studies that investigated the 2-back task [26, 38]. These studies reported similar bilateral effects localized to the inferior/ventrolateral PFC. Our approach of using 3D digitizer data and projecting optode positions to the MRI brain thus confirm findings of earlier studies regarding the spatial extend of NIRS signals induced by a n-back WM task.

Complementing knowledge on caffeine’s well-known effects in reducing resting cerebral perfusion [9, 12], the present study shows differential effects on the relative concentrations of both hemoglobin species in the course of a prolonged cognitive task. The high sampling rate of the NIRS technique allowed us to visualize dynamic changes in the concentrations of both hemoglobin species. In fMRI, repetition time (TR) is usually an order of magnitude lower and does thus not allow a visualization of these dynamic changes. The intake of 200 mg caffeine lead to a significant decrease of the HbO response between 20 and 40 s after the onset of the 2-back trial in the bilateral IFC accompanied by a corresponding increase of HbR in the left IFC. The present study thus replicates the results of Dodd et al. [39], who found similar effects of caffeine across a range of cognitive tasks. Decreased HbO coupled with increased HbR thus appear to be hallmark effects of caffeine during cognitive tasks. Apart from the aforementioned report, only a few studies have used NIRS to investigate the effect of caffeine on hemodynamic responses during cognitive tasks so far. Kennedy and Haskell [2] examined the effects of caffeine in a cognitive test battery and found a general decreased total hemoglobin (HbO plus HbR) concentration under its influence. Higashi et al. [40] measured responses over the IFC and reported reduced resting HbO, the best indicator of regional cerebral blood volume, induced by caffeine but, contrary to the present study, a similar increase in response measured while subjects performed an arithmetic test (c.f. [11], see below). Similar to the present study, Niioka and Sasaki [41] found caffeine to reduce concentrations of oxygenated and total hemoglobin on the left hemisphere when subjects performed a modified Stroop task. Similar to Li et al. [11], in the 2-back conditions, we did not find caffeine-related differences in the stimulation-induced response of HbO in tw1. In tw2, however, caffeine’s effects became evident as the relative concentration of HbO quickly decreased after the end of the 2-back task. Effects of caffeine on the decrease of the late but not the early course of the vascular response, such as in the present study, have also been found by Liu et al. [10] in fMRI research. To this end, it is well known that the BOLD response correlates with both HbO and HbR (see [42], for review). By using NIRS to dissect the BOLD response measured by other studies into the crucial determinants HbR and HbO, we show that both increases and decreases in the aforementioned parameters, respectively, may underlie caffeine-induced changes observed in fMRI studies. However, as we did not include an fMRI session in the present study, it is unclear whether these changes reflect the same mechanisms. The absence of a statistically significant effect between the relative concentrations of HbR on the right hemisphere might be related to the well-known finding that verbal WM processes generally place a higher load on left-hemispheric compared to right-hemispheric regions (c.f. [43]). Reduced concentrations of bilateral HbO thus appear to be a general vasoconstrictive effect of caffeine. Higher concentrations of HbR in the left IFC after caffeine consumption might indicate influences on the neuronal metabolic demands such as the cerebral metabolic rate of oxygen (CMRO), as we did not find differences in oxygenated hemoglobin between 2-back conditions in tw1. Indeed, an fMRI investigation shows that caffeine decreases the CBF/CMRO ratio in both motor and visual tasks [44]. With respect to contrasts between the 0-back and the 2-back conditions, unlike fMRI studies (e.g. [34]), we did not find significant load-sensitive changes. This may be due to the heightened between-subject variability in NIRS studies, resulting in lower statistical power of NIRS compared to fMRI group studies [45].

Finally, there are also some limitations to the present results which should be clearly stated. As we wanted our optode montage to be exactly in the same place during the whole experiment to evaluate the spatial extent of changes induced by the WM task, we decided against two separate experimental sessions. Thus, in our experiment, the pharmacological control condition always preceded the caffeine condition, as we also wanted to avoid contamination of the control condition with residual caffeine, which would have occurred if we had randomized the order of the different sessions. This confound makes it difficult to separate the effects of caffeine from those of repeated task completion. The observed effects could thus also be influenced by changes in motivation or by a shift in strategy use associated with repeated task completion. Also, we did not blind the different sessions or try to conceal our intention by using placebo pills at the beginning of the session. Given the strong, well-known subjective experience of caffeine ingestion in most participants, this would probably not have been successful. Furthermore, we only asked subjects to abstain from drinking coffee on the morning of the experiment. However, it has recently been shown that l-theanine, a substance found in tea, can counteract caffeine’s vasoconstrictive effects as well [39]. As we did not control for this or other dietary confounds, we cannot exclude the influence of l-theanine or other dietary factors (e.g. vitamins, see [46]) on the present results. Also, as we only restricted the intake of coffee, our results might have been confounded by caffeine intake from other sources such as tea or soft drinks.

## Conclusions

The present study shows dynamic alterations in the content of oxy- and deoxhemoglobin in the left and right inferior frontal cortices during a WM task induced by caffeine. In summary, there are three conclusions to be drawn from the present study. Firstly, caffeine had no effect on working memory performance, only a beneficial effect on simple motor responses. Secondly, effects of caffeine of brain vasculature can be detected as general reduction of HbO. Finally, the neuronal effects of caffeine are reflected in an increased concentration of HbR in the left hemisphere when performing a memory task.
